# Medical Baths for Invalid Soldiers

**Published:** 1915-04-10

**Authors:** 


### The Hospital

The Workers' Newspaper of Administrative Medicine and Institutional Life, Administration, National Insurance and Health.

No. 1503, Vol. LVIII. SATURDAY, APRIL 10, 1915. PRICE ONE PENNY

### MEDICAL BATHS FOR INVALID SOLDIERS.

War, especially when waged on the gigantic scale of the present contest, includes events of such highly dramatic quality that its routine, monotonous, and uninspiring duties are apt to be largely forgotten. This truth applies to the medical as well as to the military sphere, and it has special force in relation to the treatment of the chronic ailments resulting either from injury or from exposure to unhealthy conditions. There is general agreement that the Expeditionary Force has been well served on the medical and surgical side, though, as was almost inevitable, so considerable and so sudden a strain revealed defects that it would be folly to ignore. Nor is there any question that the efficiency of the hospital and operative methods has shown a high level of attainment in those responsible for their conduct. There is now arising a later consideration—namely, whether adequate arrangements exist for the care of convalescents and for the restoration of activity to soldiers more or less maimed or crippled by wounds or chronic ailments. In a recent number we drew attention to the important part likely to be occupied in this direction by massage and mechano-therapeutics as applied at one of the hospitals in France. A recent discussion at the Royal Society of Medicine gives us the opportunity to consider what resources towards the same end are available at home.

The facts to be faced may be stated in a brief sentence. In growing numbers, and certain to increase, are soldiers who are invalided after operations, and others who are rendered useless for the purposes of the Army by strains and other injuries to joints, and by such conditions as rheumatism, fibrositis, and more or less allied conditions. All of these have suffered in the common cause, and to all alike is due a full measure of patriotic gratitude. Even from the mere medical standpoint such cases are those of sufferers who demand assistance not less than their fellow-victims who undergo more dramatic injuries. There remains, too, the strictly utilitarian consideration, that a proportion of such sufferers will be able, if properly cared for, to return to active service in the firing-line. The medical help required will not bring those who practise it either personal glory or even public attention. Nevertheless, the work has a patriotic not less than a professional sanction.

We may assume as generally admitted that, for conditions such as have just been enumerated, the most efficient methods of treatment are of the mechanical order, and that among them properly applied baths occupy a prominent place. On the means of this character which are available the discussion at the Royal Society of Medicine gives us some useful information. At Bath the city authorities at the commencement of the war placed the bathing establishment unreservedly at the disposal of the War Office for officers and soldiers invalided home from foreign service; and the Mineral Water Hospital undertook to accommodate 250 patients. The Devonshire Hospital at Buxton offered 150 beds and free treatment to all soldiers and sailors who needed it; and similar offers of assistance were made by other places. In some measure, but by no means to anything like a full degree, these opportunities have been utilised, and with great advantage to many individual sufferers. It seems desirable that steps should be taken to apply much more widely measures which must be needed on a constantly increasing scale. To get these patients well and to get them well quickly is an object of very great importance.

The initial difficulty is to keep the War Office in its official capacity fully informed of the value of baths and allied therapeutic measures. In this country the Army authorities, it appears, do not include the hospitals and other institutions where such measures are practised among their recognised methods of procedure, whereas in many Continental countries the association between the two is a very close one. Burdened with work and responsibility the authorities can hardly be expected to move easily to a new departure, but they ought to be pressed to do so. Surgeon-General M. W. Russell, who spoke in the discussion at the Royal Society of Medicine, advised that an official statement of the resources available and of the conditions these are likely to benefit should be prepared and should be circulated widely to the various military hospitals. This is an eminently practical suggestion, for what is needed is not merely the deposit of facts and figures in the War Office, but the extension of all such information to individual medical officers charged with the care of individual patients. It is from these that proposals for balneological and mechanical treatment must come, and until they are informed no steps can be taken. In addition to the welfare of the sufferers themselves it must be remembered that the kind of patient who is here contemplated is likely to make but little progress under ordinary hospital treatment. They thus become sources of discouragement both to themselves and to the medical officers. In addition, these cases run a very chronic course, and hence are apt to remain occupants of beds which might well be devoted to other more acute conditions.

